# Atypical Carcinoid Neuroendocrine Tumor of the Ureter: A Case Report and Literature Review

**DOI:** 10.1055/s-0038-1673331

**Published:** 2018-10-09

**Authors:** Huay Shan Yuen, Gerald Henner Rix, Soumadri Sen, Venkata Ramana Murthy Kusuma

**Affiliations:** 1 Department of Urology, Colchester Hospital University NHS Foundation Trust, Colchester, United Kingdom; 2 Department of Pathology, Colchester Hospital University NHS Foundation Trust, Colchester, United Kingdom

**Keywords:** atypical, carcinoid, neuroendocrine tumor, ureter

## Abstract

Neuroendocrine tumors (NETs) of the ureter are rare, with less than 40 cases described in the literature. A majority of tumors described are poorly differentiated tumors with a poor prognosis. We present the case of a moderately differentiated atypical carcinoid NET of the ureter with a good postoperative outcome. A literature review was also performed to identify similar cases to compare their management and postoperative outcomes.

Neuroendocrine tumors (NETs) of the ureter are very rare and represent less than 0.5% of urinary tract cancers. 1 NETs can occur in any part of the urinary tract including the kidney, ureter, bladder, and prostate. Majority of NETs of the urinary tract will present in the bladder, and primary ureteric NETs are extremely rare. 1 Due to their rarity, the clinical presentation and origins of these tumors are poorly understood. 2

Less than 40 cases of neuroendocrine ureteric tumors have been reported in the literature, with the vast majority being poorly differentiated NETs. These tumors commonly present as aggressive tumors that metastasize early, leaving patients with a poor prognosis.

We present the case of a moderately differentiated neuroendocrine carcinoma of the ureter with a good outcome postoperatively.

## Case Description

**Fig. 1:**
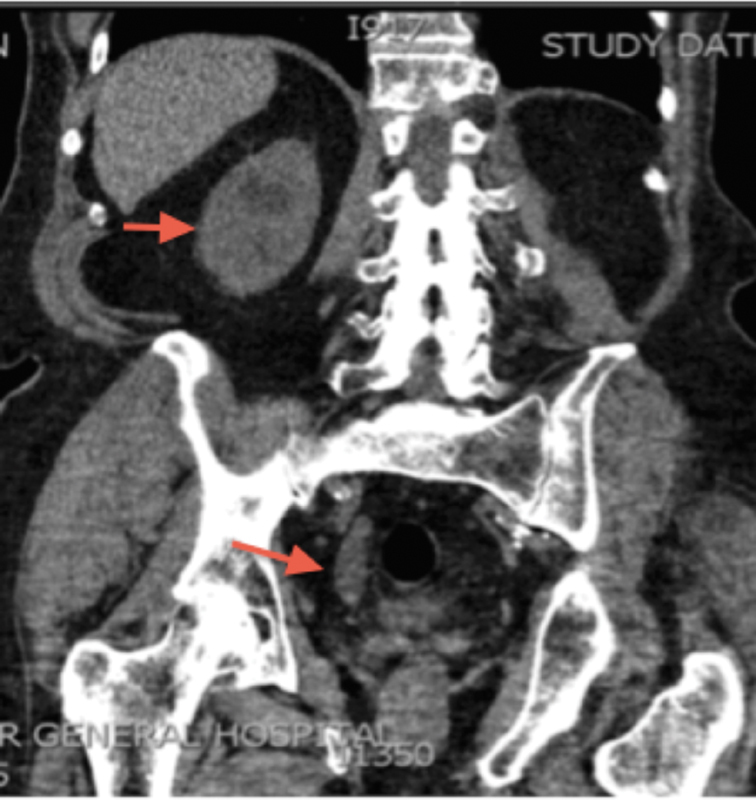
Coronal section: computed tomography of kidneys, ureters, and bladder showing the hydronephrotic kidney and dilated lower end of the ureter with narrowing at the vesicoureteric junction (arrows).

An 88-year-old man presented with right-sided loin pain and microscopic hematuria, without any urinary symptoms. Routine blood tests revealed derangement of his renal function. An initial ultrasound scan of his urinary tract revealed right-sided hydronephrosis. A further computed tomography of kidneys, ureters, and bladder scan revealed dilatation of the ureter up to the vesicoureteric junction with an associated tight stricture ( Fig. 1 ). He subsequently had a right rigid ureteroscopy, ureteric biopsy, and ureteric stent insertion. The initial histology was reported as transitional cell carcinoma of the ureter. No neuroendocrine markers were performed at the time, as the tumor did not show any classical signs of a carcinoid tumor or a well-differentiated small cell carcinoma. As demonstrated later, the histology revealed an atypical carcinoid pattern. Further imaging of his chest did not reveal distant metastases and this man underwent a right laparoscopic nephroureterectomy and open excision of bladder cuff.

**Fig. 2:**
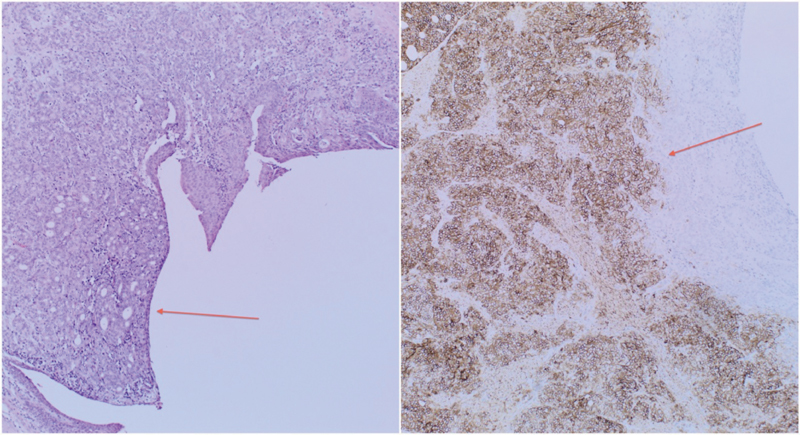
(Left) Neuroendocrine tumor from luminal side of ureter with glandular formation (arrow). (Right) Positive stain with CD56 (arrow).

**Fig. 3:**
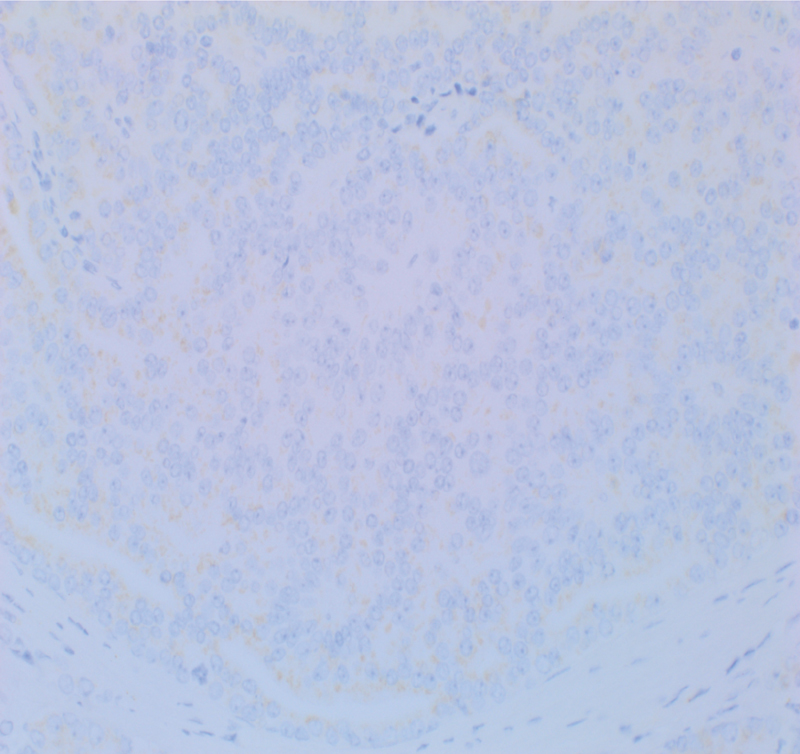
Chromogranin A staining.

**Fig. 4:**
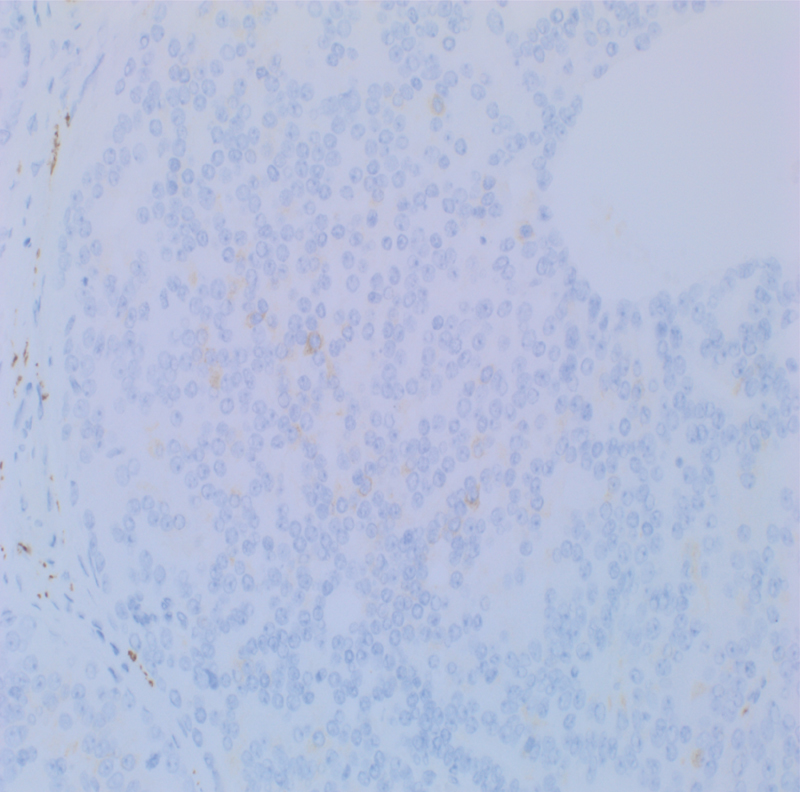
Synaptophysin.

**Fig. 5:**
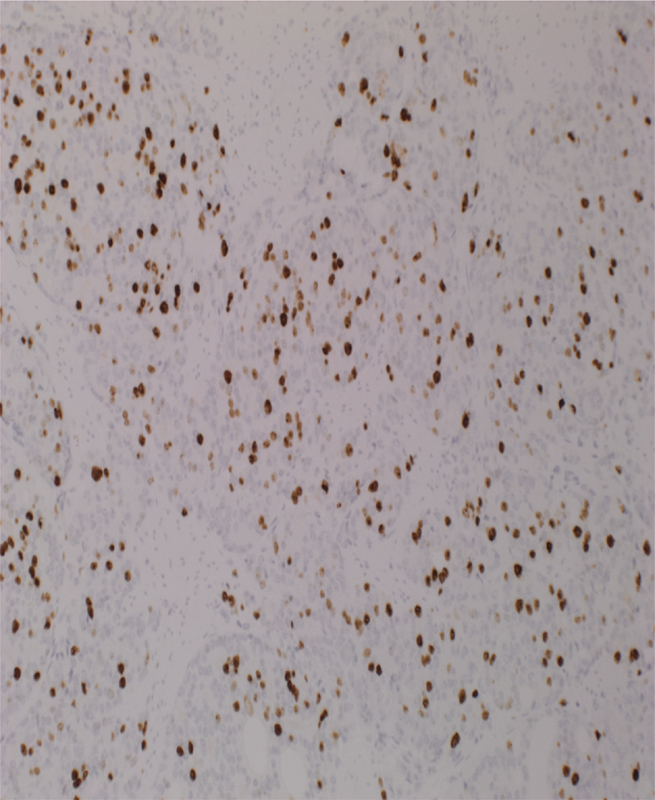
Ki-67 staining 25 to 30% positivity.

Macroscopically, the ureter showed a tumor obstructing the lumen toward its distal end, covering a length of ∼18 mm and the resection margin was clear by at least 4 mm. Microscopy of the ureteric specimen revealed a positive immunohistochemical stain with CD56, a common neuroendocrine marker ( Fig. 2 ). Staining with other neuroendocrine markers was weakly positive ( Figs. 3 and 4 ). The tumor was of intermediate to high grade, with a high Ki-67 proliferation index of 25 to 30% ( Fig. 5 ). Associated carcinoma in situ (CIS) was not seen. There was no definite lymphovascular invasion. The tumor infiltrated through the muscle into the periureteric fat (T3). The background kidney displayed patchy lymphocytic infiltrates in keeping with mild interstitial nephritis. There was no tumor infiltration into the kidney. The urothelium covering the renal pelvis did not show any evidence of CIS or tumor.

The patient made an unremarkable recovery. As he remained well and chromogranin levels were only mildly elevated and stable, the oncologist decided there was no need for adjuvant therapy. Over the past 12 months, his cystoscopies have not shown any signs of recurrence. He also had a repeat computed tomography of his chest, abdomen, and pelvis 12 months postoperatively which did not show any signs of recurrence.

## Discussion

NETs of the ureter commonly present in the sixth decade. 3 Presenting symptoms are not dissimilar to other urothelial carcinomas and majority of patients initially present with flank pain and hematuria ( Table 1 ). Immunohistochemical staining with stains such as chromogranin, synaptophysin, CD56, or Ki-67 is the key to diagnosing these tumors. Tumors are variably positive to these stains.

**Table 1:** Selected articles reporting cases of neuroendocrine tumors of the ureter

	Case	Age/gender	Presenting symptoms	Site of tumor	Management	Pathology	Follow-up (mo)	Adjuvant treatment	Outcome of disease
1	Chuang and Liao (2003) 4	57/M	Hematuria/pain	Ureter	Neph-uret	Small cell	17	Nil	Death from disease
2	Chuang and Liao (2003) 4	50/M	Hematuria/pain	Ureter	Neph-uret	Small cell	>55	Nil	No recurrence
3	Lee et al (2006) 5	70/F	Malaise	Ureter	Neph-uret	Atypical carcinoid	36	Nil	No recurrence
4	Sakuma et al (2008) 6	73/F	Hematuria	Ureter	Neph-uret	Carcinoid	9	Nil	CT not done due to comorbidities. Death from disease
5	Masui et al (2008) 7	69/M	Hematuria	Ureter	Neph-uret + bladder cuff	Small cell	14	CT (irinotecan, etoposide, cisplatin) RT	No recurrence
6	Banerji et al (2008) 3	55/M	Flank pain	Ureter	Neph-uret + bladder cuff + removal of nodes	Small cell	Not mentioned	CT (gemcitabine +carboplatin)	Not mentioned
7	Kozyrakis et al (2009) 8	78/M	Hematuria	Ureter	Neph-uret + bladder cuff incision	Small cell	6	Nil	CT not done due to comorbidities. Death from disease
8	Oshiro et al (2013) 9	78/M	Incidental	Ureter	Neph-uret + partial resection of bladder	Large cell	9	Nil	No recurrence
9	Ping et al (2014) 1	65/F	Flank pain	Ureter	Neph-uret	Small cell	4	CT (irinotecan + cisplatin)	Tumor remains stable
10	Jang et al (2013) 10	59/M	Hematuria	Ureter + bladder	Neph-uret + bladder cuff resection	Small cell	10	CT (etoposide + cisplatin) RT	No recurrences
11	Osaka et al (2015) 11	70/M	Flank pain	Ureter	Neph-uret	Small cell	38	CT (cisplatin + irinotecan)	No recurrences
12	Wang et al (2016) 2	69/M	Flank pain+ hematuria	Ureter	Neph-uret	Small cell + atypical carcinoid	12	Nil	Pt declined CT/RT. Death from disease
13	Beddok et al (2016) 12	80/M	Hematuria	Ureter	Neph-uret	Small cell	16	CT (carboplatin + etoposide) RT	In remission

Abbreviations: CT, chemotherapy; Neph-uret, nephroureterectomy; Pt, patient; RT, radiotherapy

Due to low incidence, the development of ureteric NETs is poorly understood. Four hypotheses have been put forward to suggest the origin of these tumors: (1) neuroendocrine differentiation of the urothelium, (2) direct origin from the neuroendocrine cells present in the urinary tract, (3) from the entrapped neural crest in the ureter during embryogenesis, and (4) from undifferentiated stem cells that differentiate toward an urothelial or squamous cell lineage. 3 13

Based on their behavior and histology, neuroendocrine ureteric tumors can be divided into the following categories: well differentiated (carcinoid), moderately differentiated (atypical carcinoid), and poorly differentiated (small cell and large cell) ( Table 2 ). These tumors can develop anywhere, with 74% of NETs originating from the gastrointestinal tract, 10% from the lungs, and the remainder from other parts of the body. 2

**Table 2:** Types of neuroendocrine tumors

Well differentiated (Benign behavior)	Carcinoid
Moderately differentiated (Atypical behavior)	Atypical carcinoid
Poorly differentiated (High-grade malignant)	Small cell and large cell carcinoma

Poorly differentiated ureteric NETs are the most malignant among these tumors. 14 The clinical course of these tumors is aggressive, and most present with metastases at the time of diagnosis, with a median survival of 8.2 months. 2 The mainstay of management in case of localized tumor is surgical removal which involves nephroureterectomy. As there are few case reports, there is no standard recommendation for adjuvant treatment. If the adjuvant treatment is used, most of the series recommend a cisplatin-based chemotherapy ( Table 1 ). In case of metastatic disease, cisplatin-based chemotherapy is the main modality of treatment.

Most cases described in the literature fall under the category, which includes small cell and large cell NETs ( Table 1 ). Characteristics of small cell carcinoma include a small cell size, scant cytoplasm, high mitotic rate, finely granular nuclear chromatin and faint or absent nucleoli, and frequent necrosis. 15 Large cell carcinomas also exhibit a high mitotic rate and necrosis. They are differentiated from small cell carcinomas by their large cell size, with a low ratio of nucleus to cytoplasm and nuclei with coarse, fine or vesicular chromatin, and/or frequent nucleoli. 9

Well-differentiated tumors such as carcinoids usually exhibit low-grade nuclear atypia, small number of mitoses, and a low Ki-67 labeling index. 15 They generally behave in a benign manner. Atypical carcinoids or moderately differentiated NETs are characterized by microscopic characteristics that are similar to carcinoids but with an increased nuclear atypia and higher mitotic activity. 2 5 In this case, there was a high Ki-67 proliferative activity of 25 to 30%. A majority of the atypical carcinoid tumors are poorly differentiated with metastasis seen in 55.6% of them. 14

## Conclusion

The optimal management of patients with NETs has not been well established due to the rarity of cases. As seen in Table 1 , a multimodular therapeutic approach is often adopted, which includes surgery, radiation therapy, and chemotherapy. There have been few documented cases of atypical carcinoid ureteric tumors, and our case has shown that early surgical management can give rise to a good clinical outcome.

Another learning point from our case is that initial biopsies of atypical carcinoid tumors can have similar properties to transitional cell tumors. It is, therefore, important to perform neuroendocrine stains to ensure correct diagnosis and appropriate management of tumors.
